# The impact of a multilevel interactive nursing quality control and audit application on nursing quality management

**DOI:** 10.1186/s12912-021-00767-0

**Published:** 2021-12-06

**Authors:** K. O. Pei-Ying, H. O. Chen-Shie, L. I. A. O. Pei-Hung

**Affiliations:** 1 Department of Nursing, School of Nursing, Cheng Hsin General Hospital and National Taipei University of Nursing and Health Sciences, Taipei City, Taiwan; 2 Department of Healthcare Administration, Asia Eastern University of Science and Technology, New Taipei City, Taiwan; 3 School of Nursing, National Taipei University of Nursing and Health Sciences, No. 365, Ming-te Road, Peitou District, Taipei City, 112 Taiwan

**Keywords:** Nursing quality control, Nursing audit, Technology acceptance intention, Multilevel interaction, Nursing

## Abstract

**Background:**

This study aims to investigate the effects of a nursing quality control and audit application (app) on the autonomous learning of nursing staff and nursing quality management by nursing supervisors. A multilevel interactive app is developed to assist nursing staff in conducting online autonomous learning and nursing supervisors in identifying problems and creating nursing quality improvement plans. The app could also present the different evaluation results of wards in visual charts for supervisors to review.

**Methods:**

A single-group pre- and post-test design was applied. Data were collected from 131 nurses between October 2019 and October 2020 to analyze the differences between nursing staffs’ willingness to engage in autonomous learning and the integrity of nursing quality improvement plan writing before and after the intervention. The structured questionnaires included open-ended questions that cover aspects of nursing quality control, the audit app, and the information acceptance intention of nurses.

**Results:**

The participants’ age and job title are negatively correlated with the app’s usability, while the ability to use 3C (Computer, Communication, and Consumer Electronics products including mobile phones and laptops) equipment is positively correlated with the willingness to use the app. Nurses’ satisfaction with the convenience of the online autonomous learning method is 92%, which indicates that the app could improve their willingness to learn. Following the intervention of the app, nursing supervisors’ satisfaction with the integrity of nursing quality improvement plan writing increased from 41 to 88%.

**Conclusions:**

Using information technology products to assist in nursing quality management in clinical practice has a significant effect on nurses’ load reduction and head nurses’ satisfaction. Multilevel interactive nursing quality control and audit apps can improve nursing staff’s willingness to learn independently, nursing quality, and the integrity of plan writing. Thus, nursing quality control and audit apps can be considered as suitable nursing quality control tools.

## Background

Medical care aims to provide safe and efficient patient-centered services, and maintaining superior nursing quality requires consistent monitoring and improvement of the quality of medical care provided by hospital staff. Therefore, the implementation of nursing quality control and audit programs is important for nursing care [1]. Currently, most hospitals in Taiwan have established audit teams under the quality management committee of the nursing department to monitor the quality of nursing care. Members of this audit team check and monitor the different nursing quality indicators listed in the quality monitoring criteria of the Central Nursing Quality Index Monitoring Program [2]. However, as most of these indicators are recorded manually, supervisors must spend weeks or even months reviewing the data due to incorrect or incomplete information. In addition, to ensure the accuracy and completeness of data collection, nurses or nursing supervisors have to spend considerable time performing audits, improving report writing, and transmitting documents, which makes these activities a large component of the nurses’ indirect care activities [3, 4]. Therefore, it is important to introduce information digitization to existing nursing practice, in order to improve nursing quality and the learning efficiency of ward nurses and ensure the effectiveness of nursing audits [5, 6].

### Nursing quality audit

To understand the impact of nursing on patient care, the American Nurses Association has developed a set of indicators that can reflect the quality of care. These include structural indicators covering the skill mix of nursing resources, process indicators covering skin integrity and nurses’ satisfaction, and outcome indicators covering nosocomial infection rate, patient injury rate, patient satisfaction with nursing services, pain management and health education, and patient care satisfaction [7–9]. The purpose of nursing quality indicators is to evaluate the effectiveness and adequacy of patient care, ensure the quality of care, establish a bridge between nurses and other professionals, and facilitate administrative management [8, 10]. As a good patient-nurse relationship can positively impact the quality of care, building patients’ trust in nursing staff is important. Studies have suggested that enhancing the quality of nursing care depends on nurses’ professional knowledge, continuous communication and coordination, alertness, and individualization, while treating patients as partners [9]. The Taiwan Quality Indicator Project 2017 proposed comprehensive nursing quality audit indicators, which cover various aspects, such as nursing techniques, nursing procedures, and nursing record documentation [11]. Alternatively, accreditation standards, as proposed by the Joint Commission on Accreditation of Healthcare Organization, clearly state that nursing departments should introduce planned and structured systems that monitor and evaluate nursing staff’s performance in looking after patients, solving problems, and properly compiling nursing records [10]. The Plan-Do-Check-Act cycle (PDCA) is an iterative strategy for nursing quality management that aims to maintain and continuously enhance the quality of care. Within the PDCA cycle, “Plan” focuses on planning, division of labor, and formulating future plans; “Do” focuses on the execution and implementation of the plan; “Check” focuses on the evaluation of the plan, as well as the drafting of an action plan when there is a gap between the planned and actual implementation; and “Action” focuses on consistent enhancement, the introduction of corrective measures, and the formulation of improvement strategies [10]. However, upon implementation, writing the contents of quality improvement PDCA reports has become a time-consuming burden for nursing supervisors [12].

### Evaluation of the effectiveness of applications and information systems

According to Maslow’s hierarchy of needs, scholars have redefined three hierarchies of consumer needs for a product: functionality, usability, and pleasure [13]. Therefore, when designing products, in addition to the basic functionality of the product, designers should improve its usability [14]. This is because once people become used to the functionality of a product, they shift their focus to its ease of use, and this holds particularly true for applications (apps), in which smooth usage and enhanced user experience can be achieved only with a well-designed interface [15, 16]. Alternatively, Venkatesh and Davis [17] established the technology acceptance model 2 (TAM2) to explain individuals’ behavioral intentions toward information systems. This model states that a user’s technological behavior, gender, and ethnicity can be validated and interpreted based on the simplest theoretical parameters. Of these parameters, social influence includes subjective norms, image, and other variables, while usability includes job relevance, output quality, result demonstrability, and perceived ease of use [18, 19].

## Methods

### Aim

The purpose of this study was to discuss the development of a multilevel interactive nursing quality control and audit app, examine the effects of nursing staffs’ willingness to engage in autonomous learning, determine the integrity of nursing quality improvement plan writing through a simplified nursing quality audit process for nurses and nursing supervisors, and propose a new TAM, that is, ATAM2 (adjustable technology acceptance model 2).

### Design

**Fig. 1:**
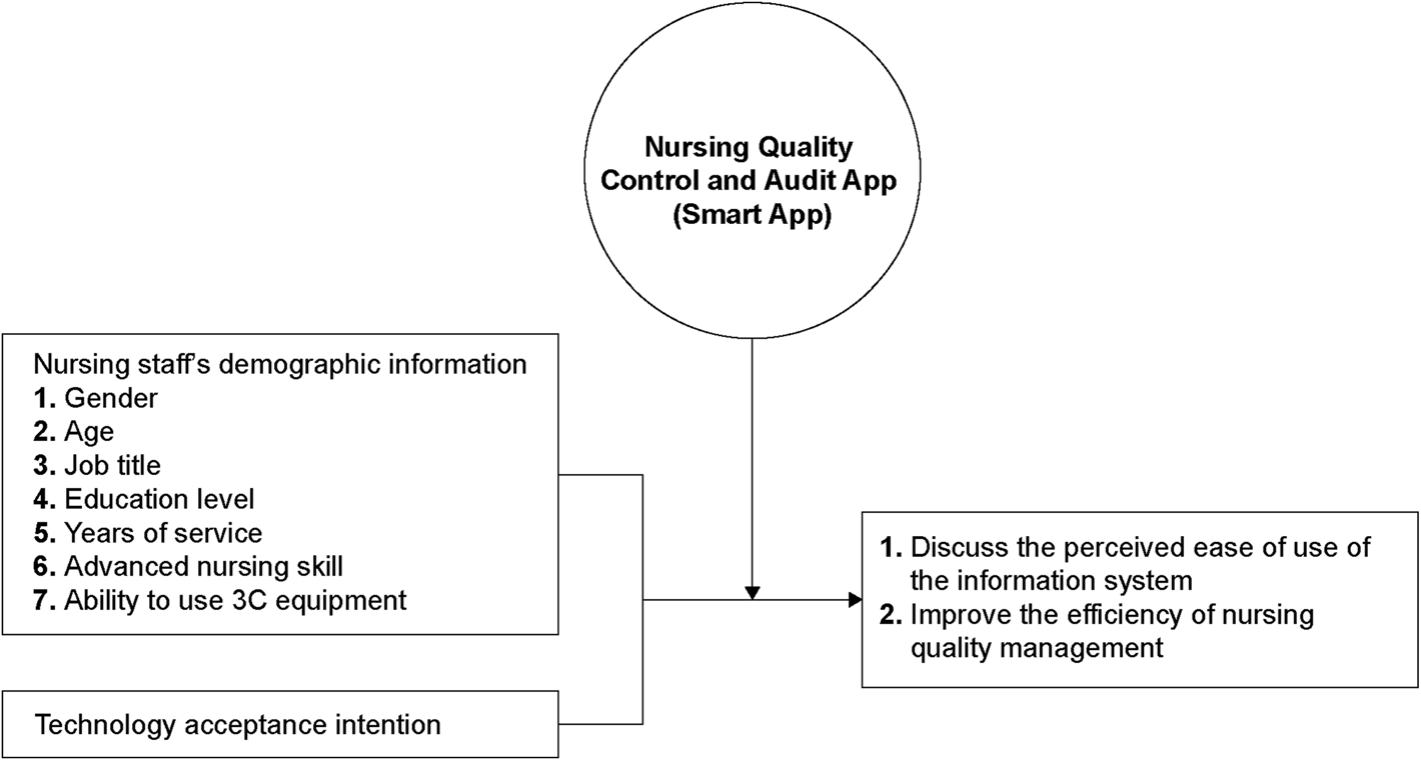
Research framework

**Fig. 2:**
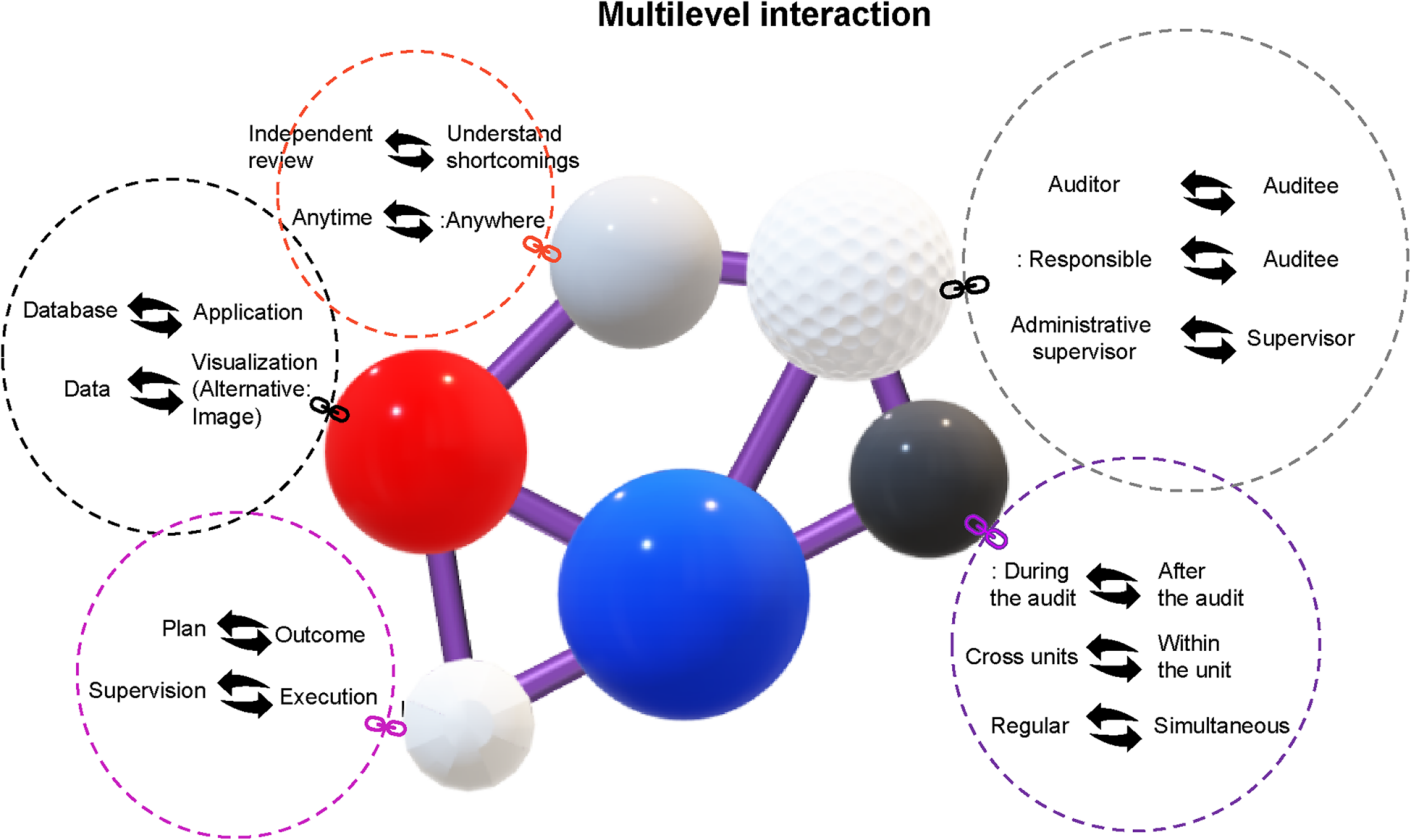
Multilevel interaction

This study adopted a single-group pre- and post-test design, and the research framework is shown in Fig. 1. Following the introduction of a nursing quality control and audit app for nurses and nursing supervisors, this study investigated the changes in nursing staff’s autonomous learning and the integrity of nursing quality improvement plan writing both before (prior to the introduction of the app) and after (6 months after the introduction of the app) the intervention. In addition, the performance of the information system was subjectively evaluated. The nursing quality control and audit mobile app were assessed using a questionnaire based on the adjustable technology acceptance model 2 (ATAM2) after 6 months of use (Fig. 1). The multilevel interaction model in this study includes nurses’ autonomous learning training, real-time communication with nursing supervisors during member assessment, reminders for nursing supervisors to write improvement plans, and integrating the achievements of each unit to senior nurses for monitoring quality. In order that the data interaction between platforms can form meaningful information as a positive cycle of improvement and supervision, the objects at all levels of the organization can interact in this real-time series. At the same time, in practice, it can be used to audit the quality of work throughout the hospital, internal education and training, and learning tests (Fig. 2).

### Setting

The study was conducted at a teaching hospital in northern Taiwan between October 2019 and October 2020.

### Participants

Calculations using G-power 3.1 (assuming power = 0.8, *α* = 0.05, effect size = 0.3 [20]) suggested that at least 90 participants should be included in this study, and at least 100 cases should be admitted to avoid the loss of participants exceeding 10%; therefore, 149 participants were initially enrolled in this study. Eighteen participants were subsequently excluded due to their contracting COVID-19, leaving 131 participants who completed the research. The inclusion criteria were: holding a Taiwanese nurse practitioner license (Registered Professional Nurse’s license), agreeing to participate in the research, using the nursing quality control and audit app, and filling in the consent form.

### Data collection

The data collection process included the signing of a consent form by the participants after they had received sufficient information about the study, indicating their intention to participate by providing a written informed consent for participation. The participants were then asked to fill in the nursing quality control and audit application questionnaire. Data collection began on October 1, 2019 and concluded on March 25, 2020, and the posttest was conducted from May 1, 2020 to October 25, 2020. The questionnaire was self-reported and all completed questionnaires were personally collected by the researchers and appropriately stored in confidentiality. In the pilot study stage, the participants spent an average of 15–20 min to complete the questionnaire. As the monitoring content of the Central Nursing Quality Index Monitoring Program consists of common nursing skills (such as hand washing, drug administration, and catheterization), neither the content of the questionnaire nor the standard of nursing care quality was adjusted for different nursing departments.

### Research tools

The research tools used in this study included two components: a questionnaire and a multilevel interactive nursing quality control and audit app. The questionnaire consisted of three parts; the first part covered basic information about the nursing staff; the second part investigated the nursing staff’s intention to accept the nursing quality control and audit app; the third part explored the nursing staff’s subjective assessment of the app’s effect on nursing quality management.

#### Basic information

This part of the questionnaire collected information about the participants’ gender, job title, age, education level, years of service, advanced nursing skills, and self-recognized ability to use 3C (Computer, Communication, and Consumer Electronics products, including computers, mobile phones, tablets, etc.) equipment.

#### Intention to accept the nursing quality control and audit smart app

After reviewing Venkatesh and Davis’s TAM 2 [17], as well as the research conducted by Wang and Xu [21], and discussing them with relevant scholars, ATAM2 was created for this study. This model includes scales for different variables (behavioral intention, contributing factors, technology acceptance, job satisfaction, and learning satisfaction) for a total of 29 questions. Questions are answered on a 5-point Likert scale, with the options of 5, 4, 3, 2, and 1 point representing “strongly agree,” “agree,” “neither agree nor disagree,” “disagree,” and “strongly disagree,” respectively. Subsequently, three nursing information experts were invited to review the appropriateness and clarity of the questions, in order that revisions could be undertaken if required. The experts concluded the total content validity index of the scales to be 0.75.

#### Development of a multilevel interactive nursing quality control and audit app

**Fig. 3:**
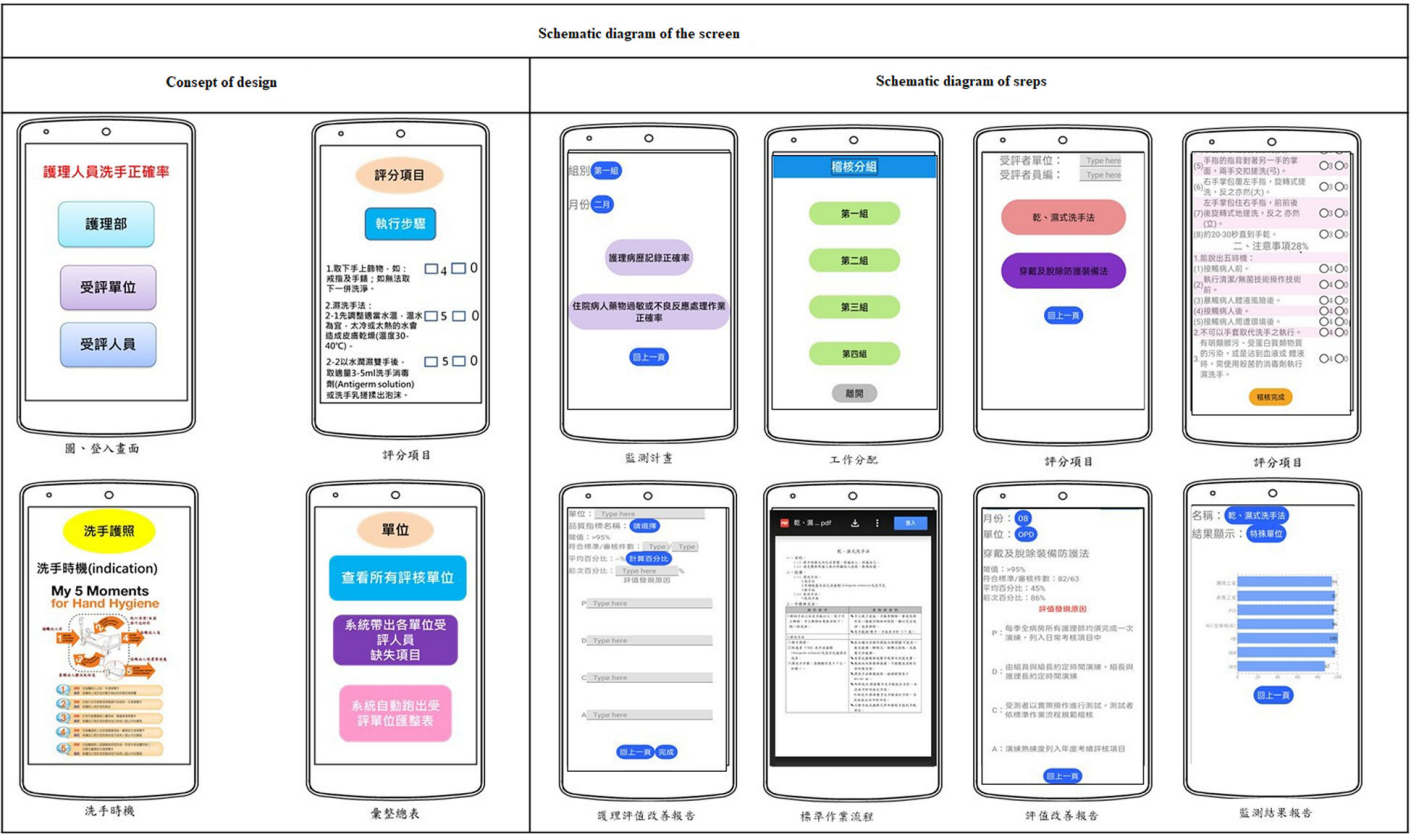
Schematic diagram of the screen

Based on previous user experience, a multilevel interactive app was designed in this study, allowing not only nurses and nursing supervisors, but individuals at all levels of the organization to interact in real-time; thus, the data communication between platforms was meaningful and could lead to a positive cycle of improvement and supervision. Additionally, the app could be used to audit the quality of the entire hospital, as well as internal continuing education, training, and real-time examinations. The contents of the app include nursing quality audit items (hand hygiene and drug delivery), online courses and videos for nursing quality audit items, courses on identifying problems and nursing quality improvement plan writing, and visual chart analysis on the compliance of different wards with audit standards. The data in the app could be mutually transmitted and simultaneously updated (Fig. 3).

**Fig. 4:**
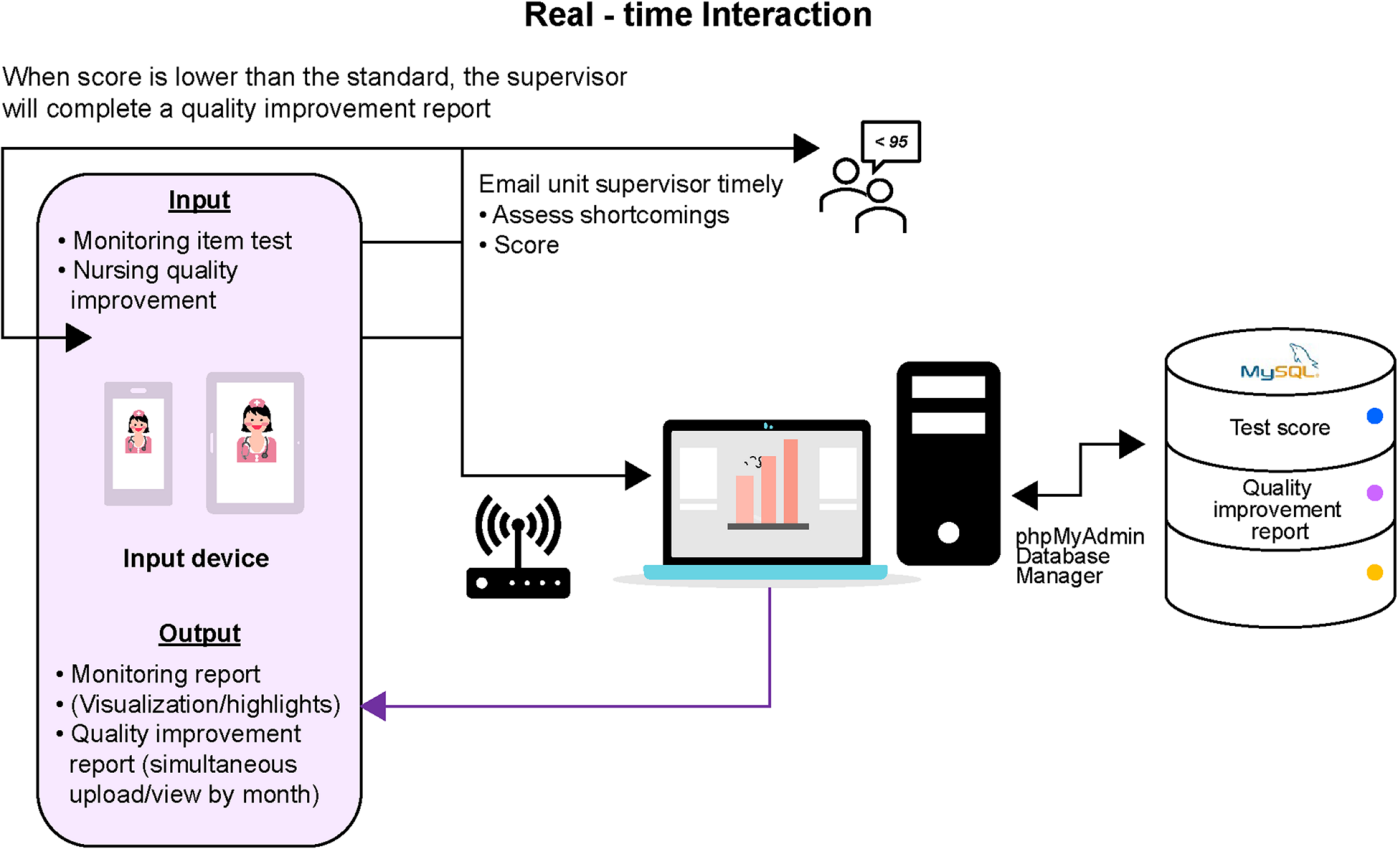
Real-time interaction system

The app was created to meet the needs of clinical users. Adopting a participatory design, the app was created by a design team composed of nursing information experts and information engineers using Thunkable X launched in June 2018. Developed by the Android studio, the app was compiled in the personal homepage program (PHP) using a programming language, and connected to the MySQLdb interface (a multi-user, multithreaded structured query language database server that effectively organizes and compiles data into tables to facilitate data query and extraction in the future) for data reception and transmission. To improve platform coverage and make the app accessible to users with different operating systems, the app was developed using Thunkable X for both iOS and Android platforms [22]. From Fig. 4, it can be seen that after the nursing standard skill assessment, members can know the items to be improved in real-time and conduct a series of autonomous e-learning. At the same time, the assessment results of the members were delivered to nursing supervisors to remind nursing supervisors that it might be necessary to write improvement plans and integrate the achievements of each unit to senior nurses for quality monitoring. Through this system, relevant personnel could understand the improvement trend of the nursing skills of each unit or member (Fig. 4).

### Data analysis

The collected data were processed using computers and data analysis methods, including mean value, correlation analysis, T-test (paired t-test), ANOVA, and other methods, which were conducted on the obtained results using statistical software IBM SPSS for Windows version 25.0 and IBM Modeler version 18.0. For example, the correlation between participants’ basic demographic information and technology acceptance intention was analyzed using Pearson’s correlation coefficient. The decision tree algorithm was adopted as the analysis tool [23, 24].

### Validity and rigor

Prior to the beginning of the experiment, the validity of the research tools was assessed (CVI:0.81) and verified by a group of experts comprised of nursing supervisors and nurses.

## Results

### Basic demographic variables and their correlations

The average age of the study participants was 37.22 (*SD* = 9.25) years, with the majority (*n* = 123, 93.9%) being women. Most participants had graduated from universities (*n* = 80, 61.1%), followed by college graduates (*n* = 45, 34.4%), and then, graduates from research institutes (*n* = 6, 4.6%). In terms of the job title, most were nurses (*n* = 57, 43.5%), followed by charge nurses (*n* = 27, 20.6%), deputy nursing supervisors (*n* = 23, 17.6%), and then, nursing supervisors (*n* = 24, 18.3%). In terms of years of service, the average was 14.379 years, with 48 (36.6%) having less than 10 years of experience, 52 (39.7%) with 11–20 years of experience, and 31 (23.7%) with over 21 years of experience. A total of 30 departments were involved in this study, with 60 participants (45.8%) from internal medicine, 19 (14.5%) from surgery, 29 (22.1%) from special wards, and 23 (17.6%) from acute wards. Regarding the classification of advanced nursing skills, nurses were categorized based on the number of years of clinical work completed: 6 (4.6%) were categorized as N (those with less than 1 year of clinical work), 34 (26.0%) as N1 (those who had completed 1 year), 47 (35.9%) as N2 (those who had completed 2 years), 36 (27.5%) as N3 (those who had completed 3 years), and 8 (6.1%) as N4 (those who had completed 4 years). There are four grades of the ability to use 3C equipment: very proficient, good, ordinary, and poor, with scores of 4, 3, 2, and 1, respectively. Most nurses believed that their abilities were between ordinary and good, and the average score of the ability to use 3C equipment was 2.47 (*SD* = 0.61). Most participants (72, 55%) recognized their ability to use 3C equipment as average, followed by 51 participants (38.9%) who recognized their ability as good.

The results suggested that age and job title were both negatively correlated with perceived ease of use, indicating that the higher the job title, the worse the perceived ease of use. Alternatively, the ability to use 3C equipment was positively correlated with willingness to use, indicating that the better the ability to use 3C equipment, the higher the technology acceptance intention. There was no significant correlation between technology acceptance intention and education level, years of nursing services, or advanced nursing skills. Analysis of covariance showed that participants with titles of nursing supervisors and nurses believed that the perceived ease of use was more important than other variables (Table 1).

**Table 1:** Correlation between basic demographics and different aspects of system usability (*N* = 131)

Variable	Usability		Perceived ease of use		Willingness to use		Contributing factors		Mobile device acceptance	
	Correlation coefficient /F (ANOVA)	P	Correlation coefficient /F (ANOVA)	P	Correlation coefficient /F (ANOVA)	P	Correlation coefficient /F (ANOVA)	P	Correlation coefficient /F (ANOVA)	P
Age	−.028	.749	−.188	.031*	−.020	.821	−.064	.470	.034	.697
Job title	/1.615	.112	/2.724	.047**	/1.628	.078	/2.486	.326	/1.882	.417
Education level	.138	.115	.086	.328	.160	.068	.086	.328	.193	.027*
Years of service	−.044	.622	−.212	.015*	−.048	.586	.339	.086	.020	.818
Advanced nursing skills	−.016	.855	.169	.053	.049	.580	.086	.330	−.019	.829
Ability to use 3C equipment	/1.112	.414	/2.428	.209	/3.601	.032**	/2.028	.224	/1.045	.258

*p < .05

**F (ANOVA test)

### Differences in technology acceptance intention before and after the intervention

The nursing staff’s technology acceptance intention before and after the intervention of the multilevel interactive nursing quality control and audit app was divided into five aspects: usability, perceived ease of use, willingness to use, contributing factors, and mobile device acceptance. Questions were rated on a 5-point Likert scale ranging from “5 = strongly agree” to “1 = strongly disagree.” The findings are as follows:

1. Participants were divided into nurses, charge nurses, deputy nursing supervisors, and nursing supervisors according to their job titles. Although there was no significant difference between participants’ technology acceptance intention before and after the intervention, an upward trend was observed in all nurses’ advanced nursing skill classification (Table 2). Alternatively, participants were divided into N, N1, N2, N3, and N4 according to their advanced nursing skills. While there was no significant difference in perceived ease of use or willingness to use, in all other variables, positive changes were noted after the introduction of the app, indicating that nursing staff’s technology acceptance intention improved. The ability advancement was compared between the two groups of (N, N1) vs. (N2 ~ N4), and there was no significant difference in perceived ease of use or willingness to use the app, which may be because the age difference between these groups is small.
2. Changes in nursing staff’s technology acceptance intention before and after the intervention were analyzed using paired t-test. The results suggest an increase before and after the intervention for usability, perceived ease of use, willingness to use, contributing factors, and mobile device acceptance. Other than the contributing factors, all other variables showed statistically significant differences after app intervention (*p* < 0.05) (Table 3).
3. The basic demographic variables of the 131 nursing staff enrolled in this study were imported into the decision tree algorithm to predict the factors influencing perceived ease of use. A decision tree is an algorithm used to establish classification models, which utilizes an inductive method to classify a series of data from top to bottom and generates a tree-like structure. A decision tree can establish a classification model using known data (training samples), and its category attributes, and predict the category attributes of new data [21]. The results showed that participants’ age, department, and education level significantly influenced their perceived ease of use, whereas gender did not.

**Table 2:** Differences in the various aspects of system usability by nurses’ advanced nursing skill classification

	N(*n* = 6)	N1(*n* = 34)	N2(*n* = 47)	N3(*n* = 36)	N4(*n* = 8)	F	P
	Mean (SD)	Mean (SD)	Mean (SD)	Mean (SD)	Mean (SD)		
Usability	3.33 (4.27)	3.11 (3.69)	4.25 (3.62)	2.80 (4.55)	1.50 (3.89)	1.249	.294
Perceived ease of use	13.83 (14.20)	11.73 (12.61)	14.44 (11.43)	16.52 (15.12)	18.87 (12.15)	.832	.007*
Willingness to use	1.66 (3.20)	2.17 (3.13)	2.74 (2.43)	2.11 (2.79)	1.37 (2.50)	.663	.019*
Contributing factors	3.00 (4.33)	2.35 (3.11)	2.78 (2.53)	1.88 (2.64)	3.37 (3.02)	.791	.533
Mobile device acceptance	2.33 (2.42)	1.67 (2.26)	2.02 (2.04)	2.05 (2.16)	1.37 (2.97)	.335	.854

*p < .0.5

Advanced nursing skill classification was as follows: N: just graduated; N1: more than one year of nursing experience; N2: more than two years of nursing experience; N3: more than three years of nursing experience; N4: more than four years of nursing experience

**Table 3:** Changes in the different aspects of system usability and user satisfaction before and after the intervention of the nursing quality control and audit app (*N* = 131)

	Before	After	T	*P*-value
	Mean (SD)/ %	Mean (SD)/%		
Usability	20.83 (3.40)	28.19 (2.54)	−9.645	.035*
Perceived ease of use	32.25 (11.21)	56.81 (6.72)	−12.842	.049*
Willingness to use	12.31 (2.54)	18.60 (1.81)	−9.513	.006*
Contributing factors	16.21 (2.48)	18.68 (1.74)	−9.986	.117
Mobile device acceptance	10.13 (1.98)	14.05 (1.30)	−10.009	.033*
Satisfaction with autonomous learning	56%	92%	7.35	.017*
Satisfaction with quality improvement plan writing	41%	88%	8.56	.002*

*p < .05

### Participants’ subjective evaluation

As the main content was to evaluate the overall APP design and user experience, text exploration was performed on the open information of the questionnaire to identify keywords in the responses. It was found that prior to the introduction of the multilevel interactive nursing quality control and audit app, the keywords were “waste,” “paper,” and “statistical difficulties,” which changed to “fast” and “convenient” after the intervention. Additionally, nursing supervisors’ satisfaction with the integrity of nursing quality improvement plan writing improved from 41 to 88%, and they felt that their workload had reduced. Participants stated that they were extremely satisfied with the visual chart analysis report and were willing to promote the use of the app in clinical practice and management (Table 3).

## Discussion

### Analysis of basic demographic data and comparison with similar studies

This study found that age and job title were negatively correlated with perceived ease of use, while education level, nursing work seniority, and nursing ability advancement were not statistically significantly correlated with overall information acceptance ability, which is consistent with the results of a previous study on the acceptance of mobile nursing stations by nurses [18]. Nurses with longer years of service perceived more difficulties with the use of the technology [25]. Alternatively, the ability to use 3C equipment was positively correlated with willingness to use. Additionally, our results indicate that young and junior nursing staff demonstrated a positive attitude toward using nursing information systems, as well as better acceptance of computers and mobiles. This finding is supported by another study, which found that senior nurses had difficulty adapting to 3C equipment, resulting in a low acceptance of nursing information systems, as well as compromised effectiveness when implementing mobile nursing stations [26]. Our study also found that nursing staff with the nursing skill classification of N4 demonstrated higher usability and willingness to use than others. This finding is contradictory to a study performed by Gao and Wu [27] which found that nursing staff with a skill classification of N showed a higher overall acceptance of mobile devices than those with a classification of N2. This difference may be due to the rapid information digitization in hospitals in Taiwan over the years; most junior supervisors, such as N4 nursing group leaders, are required to improve their digital ability, which leads to the contradictory results between this study and previous studies by Gao and Wu. Another reason for the better technology acceptance among newcomers may be due to their lack of experience with the handwriting processes in the clinical setting.

### Differences in nursing staff’s intention to accept the nursing quality control and audit app

This study used the decision tree algorithm to analyze differences in nursing staff’s technology acceptance intention after the intervention of the multilevel interactive nursing quality control and audit app. The results indicated that the ability to use 3C equipment, job title, and advanced nursing skill classification were all positively correlated with willingness to use. Particularly, the effects of better ability to use 3C equipment or a higher job title were substantial on willingness to use, which is consistent with findings of other studies [28, 29]. Moreover, following the intervention, all aspects of participants’ technology acceptance intention showed an increase, and other than that of the contributing factors, these increases were statistically significant. Therefore, it was concluded that the introduction of the nursing quality control and audit app substantially increased users’ technology acceptance intention, which is consistent with the results of another study [30].

### Influence of the intervention of the multilevel interactive nursing quality control and audit app

During this study, due to information security issues, the public hospital internet network was used to test the nursing quality control and audit app. For this reason, it was found that nursing staff experienced problems, such as network disturbances and computer crashes, when using the mobile nursing station between 8 and 9 am and 11 am–12 pm. However, according to the results of the open-ended questions, these issues did not affect the acceptance of the nursing quality control and audit mobile device, which is consistent with the results of a study performed by Chao and Lee [25]. Additionally, nursing supervisors showed increased satisfaction with the integrity of the nursing quality improvement plan writing. They felt that their workload was reduced and reported that they were extremely satisfied with the visual chart analysis report, which is consistent with the findings of a study conducted by Kaipainen et al. [1].

### Other findings

**Fig. 5:**
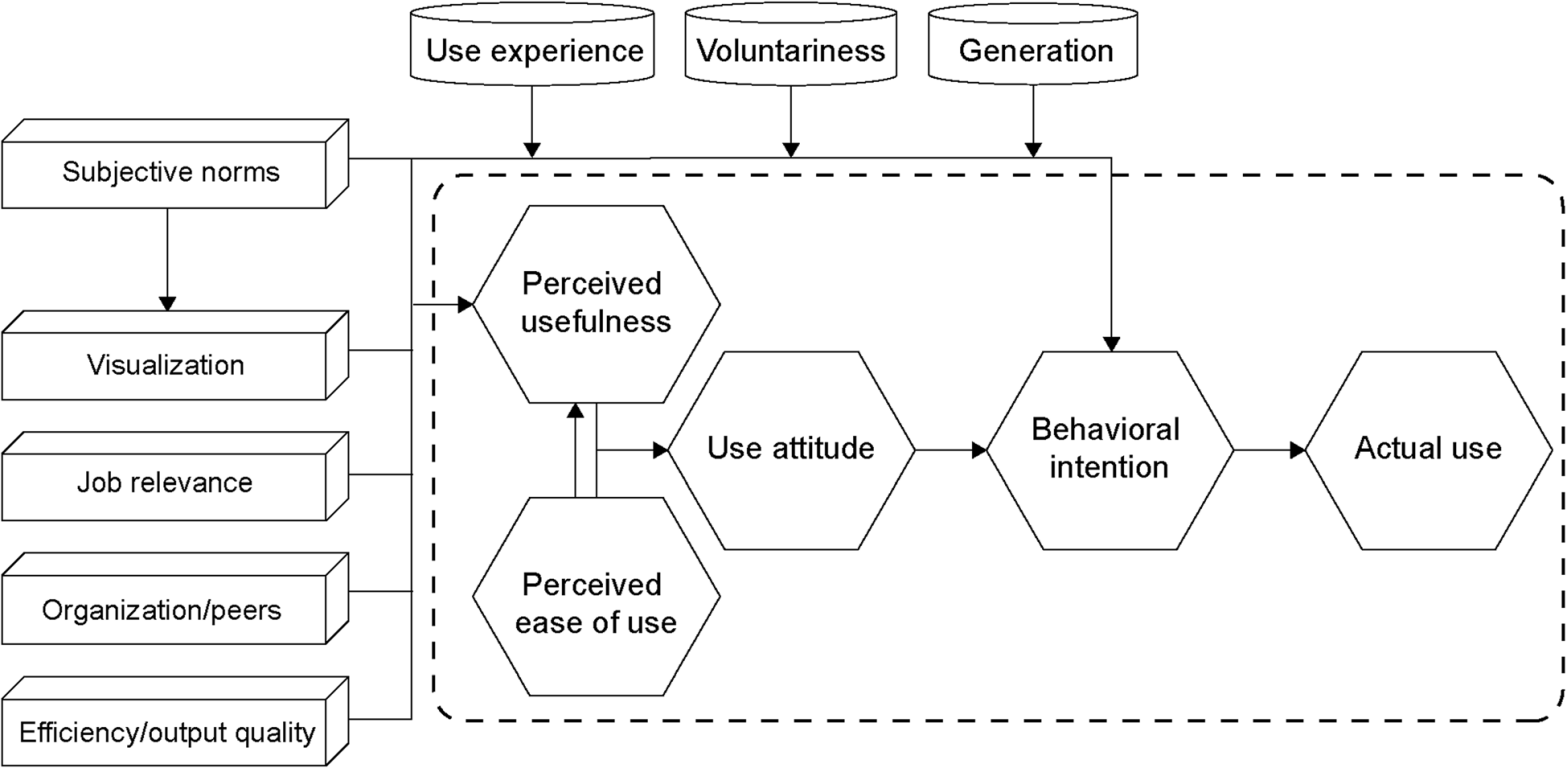
Adjustable technology acceptance model 2 (ATAM2)

With reference to the Theory of Planned Behavior (TPB) model [31], this study proposed a new TAM model, that is, ATAM2. In this model, usability and ease of use were affected by the variables, including subjective norms (such as user experience), visualization, job relevance, organizations or peers, efficiency or output quality, and result demonstrability, whereas subjective norms affected willingness to use. These findings are consistent with those of another study [32]. Furthermore, it was found that experience, voluntariness, and generational changes were interference variables for willingness to use. This is because, for users who have insufficient experience or are reluctant to use the system, subjective norms will significantly affect their willingness to use it [29]. Alternatively, in addition to behavioral changes, generational changes regarding the perception of technology will significantly affect usability (Fig. 5).

### Limitations

During validation of the TAM model for the care quality control app, we were unable to use all aspects of the original scale for measurement. Therefore, the multilevel interactive nursing quality control and audit app should be further assessed and a better-suited, revised model should be developed.

## Conclusion

This study developed a multilevel interactive nursing quality control and audit app, and examined the impact of simplifying nursing quality audit processes on nurses and nursing supervisors, and found that generational changes, age, and willingness to use the app could establish the technology acceptance model. The app could help increase nursing supervisors’ satisfaction with the integrity of nursing quality improvement plan writing, thereby reducing their workload. The visual chart analysis report could enhance nursing staff’s willingness to use the app and make behavioral changes. In addition, while age was a decisive factor in the use of 3C equipment, this study did not explore whether information learning literacy is independent of the level of work experience or whether there is a correlation between information learning literacy and work experience. The application of a quality monitoring improvement process (i.e., PDCA) enables auditors to conduct self-study and self-examination during the interactive process of nursing quality control audits, which can also enhance their learning motivation.

## Data Availability

The data that support the findings of this study are available from the corresponding author, upon reasonable request.

## Abbreviations

3C: Computer, Communication, and Consumer Electronics products, including: computers, mobile phones, tablets, etc.
PDCA: Plan-do-check-act
TAM2: Technology acceptance model 2
ATAM2: Adjustable technology acceptance model 2
PHP: Personal homepage program
